# Exploring Hmong Shamans' Beliefs on Cancer

**DOI:** 10.1002/pon.70551

**Published:** 2026-08-25

**Authors:** Ya Yambao Yang, Mandy Yang, Nancy Burke

**Affiliations:** ^1^ Department of Public Health Sciences University of California Davis California USA; ^2^ Health Occupations Preparation Butte College Oroville California USA; ^3^ Public Health Department University of California Merced California USA

**Keywords:** cancer treatment, cultural practices, Hmong shaman, shamanism

## Abstract

**Background:**

Cancer incidence among Hmong Americans has increased significantly since the early 2000s. Familiarity with and understanding of the disease is low due to limited exposure in countries of origin, lack of terminology, reliance on shamanic healing practices, and restricted access to screening. Health and healing within Hmong culture have been the domain of shamans, whose practices focus on spiritual rather than physical ailments. To understand how Hmong shamans conceptualize cancer, this study explores their beliefs, experiences, and views on collaborating with allopathic practitioners to care for patients.

**Methods:**

Fifteen Hmong shamans in the United States, Thailand, and Laos participated in semi‐structured, in‐depth interviews conducted in person or by phone. Interviews were analyzed using inductive and deductive thematic analysis.

**Results:**

Four themes emerged from the thematic analysis: beliefs about cancer's existence prior to resettlement, understandings of cancer causation, experiences with diagnosis and treating cancer patients, and perspectives on collaboration with allopathic health practitioners.

**Conclusions:**

The findings show that Hmong shamans attribute cancer to both spiritual and physical causes. While their views differ on the historical presence of cancer, there is general receptiveness to collaboration with allopathic practitioners. Future research should examine how culturally grounded models of collaboration between shamans and allopathic practitioners can be developed to improve cancer care within Hmong communities.

## 1 Introduction

Hmong people have endured centuries of displacement, with many resettling as refugees following political conflict and persecution [1, 2, 3]. Their involvement in the Secret War, a covert military operation in Laos during the Vietnam War [1], was marked by CIA recruitment and coercion to combat communist forces. This period triggered a major migration to Thailand, the United States, France, Australia, and Canada, although some remained in Laos [4, 5]. As a result of this migration history, Hmong communities are now spread across Laos, Thailand, and resettlement countries such as the United States. An estimated 330,000 Hmong people live in the United States, approximately 590,000 in Laos, and about 125,000 in Thailand [6]. Despite resettlement, Hmong shamans continue to play a vital role in community well‐being [7]. Unlike physicians, shamans address spiritual causes of illness, often identifying soul loss as the root problem [7]. Their practice encompasses spiritual and mental health disorders, infertility, injuries, and chronic diseases like diabetes and cancer [8, 9].

Within their communities, Hmong shamans have long served as respected healers. They are typically believed to be destined from birth to become shamans and to assume this role following a spiritual calling, often manifested through an illness or distress [10, 11]. Being born as a shaman means that the individual's soul is chosen and endowed with ancestral spirits [11]. In Hmong shamanism, illnesses among community members are believed to arise from spiritual disruptions such as soul loss (*poob plig*), the capture of the soul by evil spirits, or disharmony among individuals, their ancestral spirits, and the surrounding environment [12, 13]. Hmong shamans diagnose and address these illnesses through ritual practices, including the use of incense (*saib xyab*) to assess spiritual disturbance, diagnostic ceremonies (*ua neeb saib*) to identify the spiritual source of illness, and healing rituals (*ua neeb kho*) such as soul‐calling or spirit negotiation that are intended to restore balance and well‐being [13, 14, 15]. Accordingly, shamans are consulted to treat a wide range of conditions, including but not limited to soul loss and mental health disorders, infertility, epilepsy, physical injuries, and chronic diseases [16, 17].

Because many Hmong historically lived in remote, mountainous regions with little access to allopathic health care, certain health conditions were not well understood [18]. Cancer, for example, became more widely recognized only after the Secret War, when resettlement in urban areas provided greater access to diagnostic services [19, 20]. To this day, Hmong language has no native term for cancer, leading to the use of “*mob khees xaws*,” a phonetic approximation of “cancer,” or “*marën*” (มะเ็รง), the Thai term for cancer, and its closely related Lao equivalent *ມະເັຮງ*—both commonly expressed in spoken Hmong as “*mob mas lees*” [21]. Considering these cultural‐linguistic realities, recent studies have documented an increase in cancer incidence within Hmong community [22, 23]. The proportion of new cancer cases among Hmong younger than 40 years (20.2%) exceeds that of other Asian American subgroups, with Micronesians (18.1%) and Melanesians (15.7%) following [24]. Colorectal cancer is diagnosed earlier among Hmong than among other Southeast Asians, with average ages of 58.9 and 63.5 years, respectively [25]. Despite the rise in cancer cases, research has not addressed how Hmong shamans respond to the disease. Examining their views and practices could inform culturally tailored cancer care strategies.

While previous studies have highlighted the cultural significance of shamanic practices, no study has investigated how shamans view cancer or the ways they treat it [11, 12, 16, 26]. Little is also known about opportunities for collaboration between Hmong shamans and healthcare providers in cancer care. Understanding Hmong shamans' perspectives is important for informing culturally responsive approaches to engaging Hmong communities in cancer prevention, screening, diagnosis, and care. Our study addresses this gap by exploring Hmong shamans' perspectives on cancer, including beliefs about its existence and causation, experiences treating cancer patients, and views on collaboration with allopathic health practitioners.

## 2 Methodology

### 2.1 Study Design

We conducted semi‐structured interviews with 15 Hmong shamans to understand their practices, beliefs, and experiences in treating patients with cancer. This study was guided by medical pluralism, an anthropological framework that recognizes that multiple healing systems, including complementary, alternative, traditional practices, or allopathic health systems, coexist within societies, and that practitioners within these systems hold distinct but interacting frameworks for understanding illness and treatment [27, 28]. This framework is relevant for examining how Hmong shamans conceptualize cancer and navigate their role alongside allopathic care. The University of California, Merced Institutional Review Board (IRB) approved this study (Approval Number: UCM2018‐107).

### 2.2 Participants Selection

We recruited participants through snowball sampling, with initial contact made in person or by telephone, facilitated by trusted networks and with additional assistance from a local Hmong community organization based in Merced, California [29]. The inclusion criteria required participants to self‐identify as Hmong shamans. We recruited participants from the United States (California, Minnesota, Wisconsin, and Alaska), Thailand, and Laos. Five Hmong shamans declined participation, resulting in a final sample of 15. No participants withdrew after participating in the interviews.

### 2.3 Data Collection

During the development of the interview guide, a Hmong linguist reviewed the questions to ensure cultural consonance and sensitivity, and the guide was pretested with two Hmong shamans. The guide included open‐ended questions about the participants' awareness of cancer, perceived causes of the disease, treatment practices, and views on collaborating with allopathic practitioners. The questions referred to cancer in general rather than specific cancer types to reduce confusion and avoid imposing unfamiliar disease classifications on the participants. Collaboration was defined as whether shamans had previously collaborated or were willing to collaborate with an allopathic health provider in treating a cancer patient.

Interviews in California were conducted in person (*n* = 6); all others were conducted by phone (*n* = 9). The interviews were conducted between June 2018 and August 2020, with each lasting one to two hours. Before each interview, participants provided informed consent. Two participants alternated between Hmong and English during the interviews, though Hmong was the primary language used. All interviews were conducted in Hmong White, the dialect spoken by the interviewer (first author, Y.Y.). All participants were also fluent in Hmong White and reported being comfortable speaking this dialect during the interview. Y.Y.'s parents briefly attended the start of each interview to establish rapport and facilitate culturally appropriate engagement, but were not present once the interview began. Y.Y. provided clear explanations of the study's objectives, procedures, potential risks, and benefits. Participants were assured that their identities would remain confidential, and all identifying details would be removed during transcription and analysis. Code numbers were used in place of names to ensure confidentiality. Their participation was entirely voluntary, and they were reminded that they could withdraw from the study at any time without consequence.

Culturally, conversations with a shaman are preceded by seeking permission from spiritual guides at the shaman's altar. However, in our study, this practice occurred only during in‐person interviews in California. Y.Y. wrote detailed field notes during and after interviews and discussed with his mentor, N.B. With participants' consent, interviews were audiotaped. No repeat interviews were conducted. The data collection was completed in August 2020; however, Hmong shamans' understandings and approaches to cancer are unlikely to have changed substantially over time.

### 2.4 Research Team and Reflexivity

The interviews were conducted by Y.Y., who was an undergraduate student at the time of data collection. N.B. is a Professor of Medical Anthropology and Chair of Public Health, and served as the research advisor. N.B. is a medical anthropologist and public health scholar with expertise in disparities in cancer prevention, treatment, and survivorship. Y.Y. is a Hmong shaman with extensive experience interacting with other Hmong shamans and had established relationships within the community prior to the study. Participants were aware that Y.Y. was a young Hmong shaman conducting research to improve community health. M.Y. is an undergraduate health sciences student. She is Hmong, a native Hmong speaker, and comes from a family that practices shamanism, giving her deep familiarity with Hmong cultural traditions. Her cultural and linguistic expertise informed her roles as a transcriber, independent coder, and contributor to the manuscript's writing. Y.Y. identifies as male, N.B. as female, and M.Y. as female.

The research team acknowledges that Y.Y.’s dual role as a shaman and a researcher may have influenced data collection and interpretation. In particular, Y.Y.’s exposure to allopathic health perspectives may have shaped assumptions about how other shamans without exposure perceive cancer. Reflexivity was maintained through regular discussions between Y.Y. and N.B. throughout data collection to critically assess assumptions and ensure findings remained grounded in participants' perspectives. In this study, cancer and soul loss were understood as distinct but overlapping concepts, and the Western medical terminology “cancer” was intentionally used in interviews to reflect the increasing presence of cancer diagnoses within Hmong communities following migration. We acknowledge that this framing may have influenced how participants discussed illness, and this consideration informed our interpretation of the findings.

### 2.5 Data Analysis

Interviews were transcribed verbatim and translated into English for analysis. We analyzed the data using inductive and deductive thematic analysis [30]. An initial coding framework was developed based on the theoretical domains. Two independent researchers (Y.Y. and M.Y.) coded each transcript and identified recurring categories. Initial coding results were reviewed in a series of iterative meetings to refine and finalize the coding scheme through consensus. Excel software facilitated data organization and management. Final categories were confirmed through an in‐person member‐checking discussion with one participant, who was intentionally selected as the only participant with postsecondary education and the ability to engage with the findings presented in English. The participant made no requests for changes, indicating alignment with their perspectives. This member‐checking process was conducted after all completed interviews had been summarized.

## 3 Results

### 3.1 Demographic Information

The 15 Hmong shamans ranged in age from 28 to 73 and had practiced shamanism for three to 65 years. Nine resided in the United States, three in Thailand, and three in Laos. Ten were female, and five were male; all were married. Most (*n* = 13) had no formal education, with one having completed secondary school and one having attained postsecondary education. See Table 1 for participants' demographics information. Four themes emerged from the interviews: beliefs about cancer's existence prior to resettlement, understandings of cancer causation, experiences with diagnosis and treating cancer patients, and different perspectives on collaboration with allopathic health practitioners.

**TABLE 1:** Demographics of participants.

Characteristics	*n*
Age range	
20–39	3
40–59	4
60+ years	8
Gender	
Male	5
Female	10
Formal education	
No education	12
Primary	0
Secondary	1
Postsecondary	1
Location	
United States (*n* = 9)	
California	4
Minnesota	2
Wisconsin	2
Alaska	1
Thailand	3
Laos	3

#### 3.1.1 Theme 1: Beliefs About Cancer's Existence Prior to Resettlement

The participants were first asked to reflect on whether they believed cancer existed in Hmong communities prior to the displacement and resettlement that followed the Secret War. Participants' responses revealed differing perspectives on whether cancer was present before resettlement or emerged only after migration.

##### 3.1.1.1 Subtheme 1.1: Cancer Existed but Was Not Widely Recognized

Six participants believed cancer had existed prior to resettlement but was not widely recognized at the time, either because such illnesses were understood as spiritual causes or because cancer was less common due to healthier lifestyles. Their interpretations reflect how their current understanding of diseases is used to explain illnesses that were not clearly understood in the past.

I think cancer existed back in Laos, but we did not recognize it. We always had stories of Hmong people dying from mysterious illnesses, which we attributed to ghosts. Maybe it was cancer, but we did not know because our shamans back then were better at addressing spiritual causes, even though they could not cure those illnesses. I believe it existed long before our time.

(P10, female, age 28, United States)

I feel that cancer existed back in Laos but was less common because Hmong people lived a healthier lifestyle and ate natural foods.

(P11, female, age 38, United States)

##### 3.1.1.2 Subtheme 1.2: Cancer as a New Emerging Illness After Resettlement

In contrast, nine participants reported cancer was unfamiliar prior to Hmong migration to Western countries. They cited lifestyle changes, environmental influences, and diet as possible causes for its emergence. This suggests that cancer is perceived as a new illness associated with resettlement rather than a historically familiar condition. Interestingly, all three of the participants from Laos believed cancer did not exist before resettlement, indicating differences in how cancer is understood based on place and lived experience.

I don’t think cancer existed back when I was younger and still lived in Laos. We are getting cancer now because of all the processed food we eat. We don’t exercise anymore and stay home every day, especially for us older people who don’t work.

(P2, female, age 73, United States)

#### 3.1.2 Theme 2: Understandings of Cancer Causation

While all participants reported familiarity with cancer, their understanding reflected a range of perspectives. Participants described cancer causation using spiritual, physical, and social explanations, often drawing on multiple explanations. Many described cancer through a Hmong cultural and spiritual framework, invoking concepts such as soul loss, depression, and fate.

##### 3.1.2.1 Subtheme 2.1: Cancer Caused by Soul Loss

Fourteen participants identified soul loss as a cause of cancer. This reflects the continued centrality of spiritual explanations in the understanding of cancer, even when participants acknowledge multiple contributing factors.

I believe the loss of a soul causes cancer. If you are depressed, then it is easy for your soul to wander around and be captured by an evil spirit. It is important to find the right shaman to treat you….

(P6, female, age 53, Thailand)

If a person has cancer, it is due to soul loss. It is crucial to diagnose the correct soul, as each person has 12 souls. Identifying where the lost soul has gone is essential. Sometimes, it is captured by a mighty evil spirit, making the diagnosis especially challenging.

(P8, male, age 65, Laos)

##### 3.1.2.2 Subtheme 2.2: Biosocial Change as Cancer Causes

Others associated the development of cancer with consumption of processed foods containing chemicals, insufficient physical activity, hereditary factors, exposure to electricity, and environmental risks such as sun exposure and water contamination. These accounts reflected an understanding that cancer has multiple possible causes.

Cancer is caused by many things. I am certain it is caused by the processed food we eat. We have these huge fruits and vegetables only possible with certain chemicals and fertilizers. We also don’t exercise that much in America. I don’t think it’s pertaining to Hmong people, but other races like to go under the sun, and I learned that it increases your chance of getting cancer. If we think from a spiritual view, soul loss causes cancer, too.

(P10, female, age 28, United States)

That is a hard question. I did not go to school, but I know a little English. My doctors told me that the cause of cancer is unknown. They said there are many reasons. Some say it is genetics. Others say it is your food. Nowadays, we eat too much processed food. Then some say it is the weather we live in. It is not good to be under the sun too much. I think there are too many possible reasons… I do not think cancer has anything to do with spirituality. I do think that our spirit has to do with the severity of our symptoms if we have cancer.

(P12, female, age 58, United States)

I think it is because we are now living in houses with electricity and using cars, which exposes us to harmful electricity, and this is causing cancer.

(P1, male, age 68, Laos)

##### 3.1.2.3 Subtheme 2.3: Misdiagnosis Among Allopathic Health Practitioners

Beyond spiritual and lifestyle factors, several shamans emphasized misdiagnosis by allopathic health practitioners as shaping how cancer is identified and understood. This reflects a shamanic perspective in which cancer diagnoses are viewed with skepticism, especially when medical explanations are unclear.

I think there are many reasons why people develop cancer. When I was growing up in Laos, we never had cancer. Sometimes, I wonder if it is a disease that doctors diagnose simply to have something to attribute symptoms to, especially when they cannot figure out what is wrong.

(P5, male, age 50, Thailand)

##### 3.1.2.4 Subtheme 2.4: Differences in Views by Generation

Participants' understandings of cancer also differed by age. All younger participants described cancer as caused by both spiritual and physical factors, whereas older participants more often emphasized spiritual causes, although some also acknowledged physical factors.

I think it’s mostly caused by our souls wandering around or being captured by evil spirits. I know it has to do with the food we eat, but that’s just a small part of it. If our souls are securely attached to our bodies, then we won’t really get cancer. It’s easy to get it if your soul has many issues and is not secured to your body.

(P3, female, age 63, United States)

#### 3.1.3 Theme 3: Experiences With Diagnosing and Treating Cancer Patients

Participants described their experiences diagnosing and treating cancer patients through ritual practices, including interpreting incense to guide diagnosis and performing healing ceremonies for treatment. Their experiences varied, with most participants describing challenges in diagnosing and treating cancer, while others reported successfully diagnosing and treating cancer through their healing practices.

##### 3.1.3.1 Subtheme 3.1: Challenges in Cancer Diagnosis and Treatment

Eleven participants described challenges in diagnosing and treating cancer, particularly difficulty determining its cause because it did not present clearly through their usual diagnostic methods. They explained that both the interpretation of incense and the performance of healing ceremonies were often unclear when working specifically with cancer patients. This suggests that cancer is understood as complex and rooted in bodily processes rather than in spiritual causes by most participants.

Usually, patients bring three incense sticks to me and place them near my altar. Then, they bow and ask for help from my spiritual guides. I look at the incense sticks to diagnose them. It is challenging to diagnose people with cancer because their illness does not appear on the incense. It seems blurry. Sometimes, I see that their soul is sad, but I know that is not the main cause of their cancer. I know that their sadness worsens their symptoms.

(P4, female, age 60, United States)

##### 3.1.3.2 Subtheme 3.2: Cancer Diagnosis Understood Through Spiritual Causes

Four participants described successful experiences diagnosing and treating cancer through their shamanic practices. They reported that their spiritual guides helped them identify the cause of a patient's cancer. These accounts indicate that cancer can have spiritual origins that require spiritual intervention, and that allopathic approaches alone may not fully address the needs of Hmong cancer patients.

I had a patient with breast cancer who came to me for help. She was told she would only live up to a year. She was crying, and I was able to help her. When I was diagnosing her through incense, I could see that her soul had been captured by an evil spirit in the forest. The evil spirit wasn’t a normal one. It was a leader who had many followers. He was very powerful. After I diagnosed her, she felt better, but it required a healing ceremony. I performed a ceremony for her, and the breast cancer tumor eventually got smaller. She also had chemotherapy right after that. I think it was both treatments that helped.

(P7, female, age 59, United States)

#### 3.1.4 Theme 4: Different Perspectives on Collaboration With Allopathic Health Practitioners

Participants described different perspectives on collaboration with allopathic health practitioners in the care of cancer patients, emphasizing both perceived benefits and challenges. They all had provided treatment for cancer patients, with care often directed toward addressing spiritual imbalances, particularly retrieving lost souls, and managing physical symptoms. Only three participants reported prior collaboration with allopathic health practitioners, each instance occurring in the United States. Their views on future collaboration varied, with some citing positive experiences and others expressing skepticism.

##### 3.1.4.1 Subtheme 4.1: Openness to Collaboration

Most participants (*n* = 9) stated that they would be willing to work with allopathic health practitioners to treat cancer patients. They emphasized shared goals of patient well‐being and the complementary benefits of spiritual and allopathic care. Five participants mentioned that they had referred patients to allopathic providers when their spiritual healing was no longer effective. This indicates that most participants see collaboration as beneficial to patient care.

One time, I had a patient who was very sick and fatigued. He was hospitalized for days in the ICU. His doctors and nurses were treating him, and his family also came to seek treatment from me. I performed a healing ceremony for him, and he woke up the day after. It was both treatments that contributed to the improvement of his health………..At the end of the day, we all share the same goal: to help our patients heal. Integrating Western medical treatments with traditional Hmong healing practices could be very beneficial, but it would require someone within the hospital system to lead that change. Doctors are the experts in understanding the body, such as organs and blood vessels, while shamans focus on the spiritual side. Collaboration would be essential to creating a truly effective treatment plan.

(P13, male, age 28, United States)

I was treating a Hmong lady with stomach cancer. I performed one diagnostic ceremony and two healing ceremonies, but she didn’t get better. At first, I thought she was possessed by an evil spirit that lives near the water, but I got rid of the evil spirit, and her pain didn’t go away. I gave up and referred her to a medical doctor. Then, she told me she was diagnosed with stomach cancer. She passed away a few years later.

(P2, female, age 73, United States)

##### 3.1.4.2 Subtheme 4.2: Reluctance to Collaborate

In contrast, six shamans expressed that they would not be open to collaboration. Their reluctances were shaped by both relational concerns and spiritual beliefs. Some attributed their reluctance to perceived disrespect from allopathic practitioners, which they believed stemmed from their lack of formal education. This suggests that challenges in collaboration are shaped by past experiences rather than by a lack of interest in collaboration.

I am not sure. I do not think so. It feels like they do not value the skills we bring because we are seen as uneducated Hmong people without a country. I do not think there is much respect for Hmong shamans.

(P5, male, age 50, Thailand)

One shaman noted that working with allopathic practitioners was unnecessary when fate had already decided the course of a person's life, reasoning that no medical treatment could change what was spiritually predetermined. This shows that spiritual beliefs about illness influence whether collaboration is considered necessary.

I believe cancer is caused by destiny. Before we are born, our lifespan is already determined. Cancer is simply another illness that fulfills the role of taking us away when we have reached the days we were destined to live. There is no point in collaborating when your destiny is already decided.

(P9, female, age 62, Laos)

## 4 Discussion

Our study found that Hmong shamans' beliefs about cancer reflect a blend of spiritual, physical, and environmental perspectives. Their historical views on whether cancer was present prior to resettlement were mixed, with nine shamans linking cancer to changes after resettlement and six stating it was absent beforehand. While all shamans had treated at least one cancer patient, their explanations for the disease varied, citing factors such as soul and fate, sedentary lifestyle, processed foods, genetics, and exposure to electricity. Most shamans described challenges in diagnosing and treating cancer patients from a spiritual perspective, while a smaller number reported successful experiences. Collaboration with allopathic providers was rare, but nine shamans expressed willingness to engage in future partnerships. The other six shamans were skeptical of collaborating with allopathic health practitioners due to past negative experiences. These results highlight that Hmong shamans have different beliefs on the causes of cancer and point to an opportunity to develop culturally informed educational initiatives that bridge shamanic and allopathic approaches to care.

Our findings are consistent with broader literature indicating that Hmong health beliefs are pluralistic, incorporating spiritual, physical, and environmental explanations for illness. Hmong people's understandings of illness have been described as involving disruptions across natural, social, and supernatural realms [31]. Our study is unique because it provides specific insight into how shamans articulate these explanations in the context of cancer. Of note, different perspectives across generations emerged, revealing differences in how younger and older shamans understand the causes of cancer. Younger shamans more often described cancer as involving both spiritual and physical factors, whereas older shamans generally attributed it primarily to spiritual causes. These differences may reflect differences in acculturation and exposure to allopathic medicine, particularly among younger shamans. Future qualitative research should explore how Hmong shamans' beliefs and practices related to cancer change over time, particularly across generations. This may offer insights into how shamanic healing knowledge is maintained and adapted within contemporary healthcare contexts.

Beyond how shamans conceptualize illness, these beliefs also shape how treatment decisions are made. A study found that Hmong individuals' decisions depended on the illness they had and whether it was considered spiritual [9]. Our findings align with this work in that Hmong shamans also distinguish between spiritual and physical illness when deciding whether care should involve a shamanic healing ceremony, allopathic health treatment, or both. However, our study differs because it shifts the focus to Hmong shamans' perspectives and experiences with choosing the appropriate care, specifically for cancer. The alignment between how Hmong patients and shamans approach treatment decisions suggests a shared cultural framework for understanding illness that is consistent with medical pluralism, where individuals navigate multiple healing systems based on the nature of the condition rather than choosing one system exclusively.

While Hmong shamanic healing has often been characterized as incompatible with allopathic medicine [8, 32], most shamans in our study reported being open to collaborating with allopathic health practitioners. This finding aligns with anthropological literature on medical pluralism, which recognizes that individuals and communities draw on multiple healing systems simultaneously, and that shamanism and allopathic health practitioners can coexist and even collaborate rather than operate separately [27, 28]. Shamans emphasized shared goals of patient well‐being and viewed spiritual and allopathic care as complementary rather than competing. Some had already taken steps toward collaboration, referring patients to allopathic providers when shamanic healing was no longer effective. Notably, all three instances of collaboration occurred among shamans based in the United States, where there may be greater institutional support for complementary healing practices and shamanism, including through federal initiatives such as the National Center for Complementary and Integrative Health [33]. In contrast, allopathic health practitioners in Laos and Thailand may have less familiarity with or openness to shamanism, and in some cases may dismiss shamanic practices as unscientific. Hmong shamans' openness to collaboration with allopathic health practitioners is promising, as such partnerships may improve the quality and cultural responsiveness of cancer care within Hmong communities.

### 4.1 Clinical Implications

Our research offers new insights into Hmong shamans' perspectives on cancer that may inform culturally responsive approaches to cancer care. To our knowledge, this is the first study to examine Hmong shamans' understandings of cancer causation, their experiences treating cancer, and their perspectives on collaboration with allopathic health practitioners. These insights highlight the importance of recognizing spiritual healing practices as a meaningful component of cancer care for Hmong patients. Culturally sensitive training for allopathic health providers to increase awareness of and respect for shamanic practices may strengthen trust and improve the care experiences of Hmong patients navigating cancer treatment. Hmong cancer patients may not be fully cared for when allopathic health professionals are unaware of or dismissive toward shamanic practices, and greater awareness of coordination with shamans could better support Hmong patients navigating cancer. Future research should explore how opportunities for collaboration between shamans and allopathic practitioners can be developed in culturally respectful ways.

### 4.2 Limitations

While our study builds on existing knowledge, the findings should be interpreted with consideration of several limitations. First, the presence of family members during the interview initiation was not included in the IRB protocol. While parents were present only briefly to establish rapport and did not participate in the interviews themselves, this reflects a broader limitation of standard IRB frameworks, which are designed around individualistic notions of autonomy and consent and may not fully anticipate culturally embedded norms of trust‐building in Hmong and other collectivist communities. Additionally, only three participants were under 40. Although shamanic practice often begins after this age, younger shamans may have been exposed to more modern health information and media, potentially shaping their perspectives on cancer. Furthermore, only two participants had completed at least secondary education. Hmong people's history of displacement and persecution has resulted in generally low levels of formal educational attainment, particularly among older individuals. Formal education level may influence familiarity with allopathic medicine. Lastly, the majority of participants (*n* = 9) resided in the United States. These shamans may have unique perspectives shaped by greater access to healthcare and exposure to Western media, which may not reflect the views of shamans in Laos or Thailand. Recruiting more participants from varied age groups, educational backgrounds, and geographic locations would strengthen future research.

## 5 Conclusion

Our study demonstrated that Hmong shamans attributed cancer to both spiritual and physical causes and had differing opinions on whether cancer was present prior to resettlement. The majority of the participants (*n* = 11) described difficulties in treating cancer, whereas four reported no such challenges. Nine of the 15 shamans expressed willingness to work with allopathic health providers to treat cancer patients. These findings indicate opportunities to integrate shamanic and allopathic medicine approaches to improve cancer care in the Hmong community. Future community‐based interventions should emphasize provider training on shamanic practices and culturally appropriate education for Hmong communities to build mutual respect and understanding between Hmong shamans and allopathic healthcare professionals.

## Author Contributions

**Ya Yambao Yang:** developed the protocol, conducted the interviews, analyzed the data, and wrote the manuscript. **Mandy Yang:** contributed to the transcriptions of the interviews, analyzed the data, and wrote the manuscript. **Nancy Burke:** developed the protocol, supervised the study, wrote the manuscript, and edited the final version.

## Funding

This study was funded by the Undergraduate Research in the Humanities Fellowship, funded by the Andrew W. Mellon Foundation, and the Summer Undergraduate Research Fellowship from the Office of Undergraduate Education at the University of California, Merced.

## Conflicts of Interest

The authors declare no conflicts of interest.

## Data Availability

The data that support the findings of this study are available on request from the corresponding author. The data are not publicly available due to privacy or ethical restrictions.
